# Supplementary material for: Electrobiocorrosion by microbes without outer‐surface cytochromes
Source: mLife. 2024 Mar 19;3(1):110–8. doi: 10.1002/mlf2.12111 (PMC11139208; doi:10.1002/mlf2.12111)
Supplement: Supplementary file 1 — Supporting information. [file MLF2-3-110-s001.pdf]

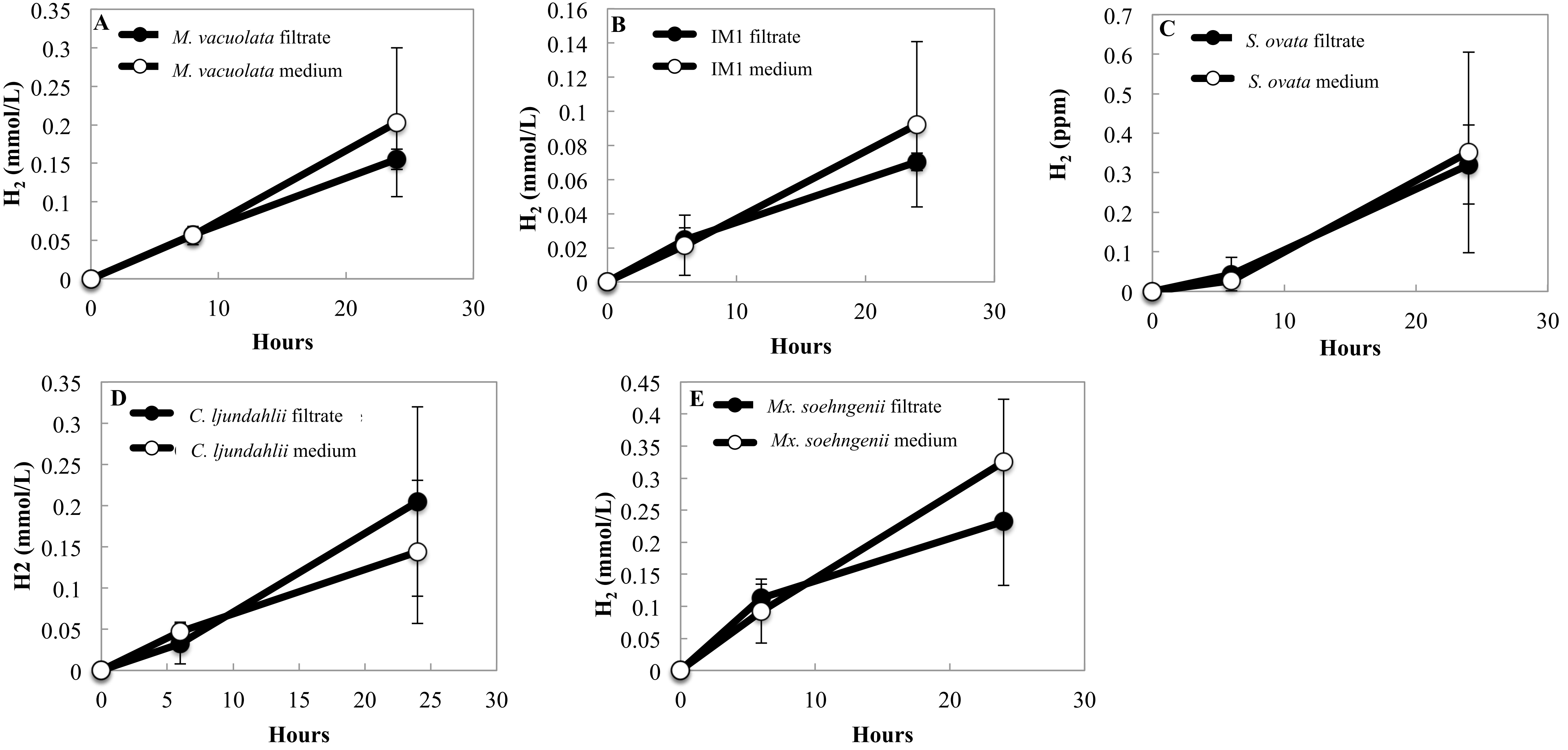

Supplementary Figure S1. H_2 production by pure Fe^0 in the presence or absence of filtered supernatant from the various cultures. For filtrate samples: one milliliter of the appropriate mid-logarithmic culture was filter sterilized and inoculated into 9 ml of medium with 2 g pure Fe^0 granules. For control samples: one ml of un-inoculated medium was added to 9 ml of medium with 2 g pure Fe^0 granules. Error bars represent triplicate samples.

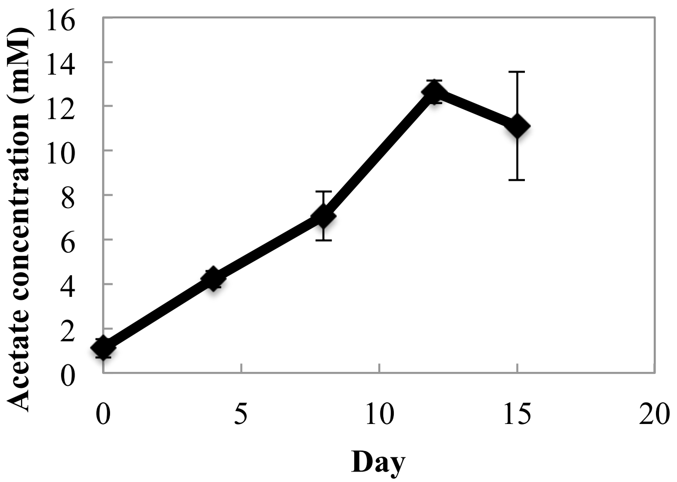

Supplementary Figure 2. Acetate generated by *Sporomusa ovata* cultures grown with H_2 (100 kPa) as the sole electron donor and CO_2 as the sole electron acceptor. The results are the means and standard deviations of triplicate cultures.
